# Lower Musculoskeletal Fitness Among Youth with Disabilities, Ages 6 to 15 Years

**DOI:** 10.3390/ijerph22020302

**Published:** 2025-02-17

**Authors:** E. Andrew Pitchford, Willie Leung

**Affiliations:** 1 School of Exercise, Sport, and Health Sciences, Oregon State University, Corvallis, OR 97331, USA; 2 Department of Health Sciences and Human Performance, University of Tampa, Tampa, FL 33606, USA; wleung@ut.edu

**Keywords:** physical fitness, disability, children, adolescents, health, secondary data analysis

## Abstract

Background: Youth with disabilities (YWD) often exhibit deficits in physical fitness, but much of the literature is limited by small, non-representative samples. The purpose of this study was to examine differences in musculoskeletal fitness between youth with and without disabilities from the 2012 National Youth Fitness Survey (NYFS). Methods: A secondary analysis was conducted with cross-sectional data from the 2012 NYFS of youth, 6 to 15 years of age. Fitness measures included plank, modified pull-ups, and grip strength. Disability was identified by multiple parent report items. Logistic regression with sampling weights was employed to examine group differences and associated factors. Results: A total of 1177 youth were analyzed, including 173 YWD. A significantly greater proportion of YWD demonstrated low fitness in all three measures compared to youth without disabilities. Factors associated with low fitness included sex, engagement in physical activity, and the body mass index category. Age was not a significant factor for any fitness measure. Conclusions: This secondary analysis provides additional evidence for lower musculoskeletal fitness among YWD, in particular for females and youth with obesity. Physical fitness continues to be an area in need of intervention to improve overall health among YWD.

## 1. Introduction

Physical fitness is an important health indicator for youth [1]. A substantial body of the literature supports that multiple components of physical fitness are directly related to improved health, including cardiorespiratory fitness, musculoskeletal fitness, and body composition [2,3,4,5,6,7,8]. In particular, musculoskeletal fitness during youth is associated with healthier weight status, skinfold thickness, metabolic indicators, cardiovascular disease risk, and bone mineral density later in life [6,7,8]. However, the importance of musculoskeletal fitness is not always reflected in guidelines for practice, with emphasis largely going to aerobic physical activity and cardiorespiratory fitness [9]. For example, the most recent physical activity guidelines from the World Health Organization [10] recommend engaging in activities to strengthen muscles and bones at least three days per week, but only after recommending that the average of 60 min per day of physical activity across the week be “mostly aerobic”. Thus, musculoskeletal fitness is an often-overlooked component of health [9].

While substantial evidence exists related to trends and outcomes associated with health-related physical fitness in the general population of youth, there is limited evidence regarding youth with disabilities (YWD). It appears that YWD exhibit lower health-related physical fitness, including musculoskeletal fitness, compared to same-aged peers without disabilities [11,12,13,14,15,16]. However, the scope of this evidence is limited by small sample sizes with limited generalizability, especially in the United States. It is important to understand whether these deficits in physical fitness are reflected in population-representative surveillance data to better inform continued surveillance, health promotion, and physical education initiatives specifically targeting YWD.

In 2012, the National Health and Nutrition Examination Survey (NHANES) included the National Youth Fitness Survey (NYFS). The 2012 NYFS had multiple measures of musculoskeletal fitness for youth [17]. There have been multiple secondary data analyses published with the fitness data from the 2012 NYFS [18,19,20,21,22,23,24,25,26]. However, differences in fitness based on disability status have not been examined. The purpose of this secondary data analysis was to examine associations of musculoskeletal fitness measures and reported disability status among youth, ages 6–15 years, from the national NYFS multi-probability sample.

## 2. Materials and Methods

This study is a secondary analysis of cross-sectional data from the 2012 NYFS. The NYFS was designed based on NHANES and used a stratified, multistage probability sampling design for a representative sample of the civilian, non-institutional resident population of the United States with youth age bands of 3–5 years, 6–11 years, and 12–15 years [17]. The National Center for Health Statistics Ethics Review Board provided human subject research approval for the 2012 NYFS procedures [17].

Among 6–15-year-old youth in the 2012 NYFS dataset, 1224 completed physical examinations [17]. Youth with valid data on the fitness measures were included in the secondary analysis. Detailed methodology for the data collection of each measure is available in the 2012 NYFS manual [17]. Low fitness on each musculoskeletal fitness measure was defined as the ≤20th percentile [21] for analysis. Status codes for “not done” were treated as missing data and excluded for complete case analysis.

The following NYFS fitness measures were included in analyses. Core muscular endurance was measured with the isometric plank test. The plank score was recorded in seconds for the time the participant held the plank pose [27]. Consistent with previous analyses of these data [25,28], a combination of “completed” and “could not obtain” status codes were entered as a plank score of 0 s. Upper body muscular strength was measured with the modified pull-up and grip strength tests. The modified pull-up score was recorded as the number of completed repetitions with correct form [29]. The grip strength score (kg) was represented as the average of repeated measurements [30]. Relative grip strength was then calculated as grip strength kg/body mass kg to normalize measurements for body size [21,31]. The body mass index (BMI) percentile was used to categorize body composition status based on measured height and weight in the NYFS protocol [32] and interpreted with the 2000 CDC sex-specific BMI-for-age growth charts [33]. Youth were classified as underweight (≤5th BMI percentile), normal weight (≥5th to ≤85th), overweight (≥85th to ≤95th), or obese (≥95th) [33].

Participant demographics were collected from a demographic questionnaire completed by a proxy respondent (e.g., parents or legal guardians). Variables used in analyses included sex (female, male), Hispanic origin (Hispanic, non-Hispanic), meeting physical activity guidelines (yes, no), and engagement in physical activity in the last seven days (yes/no) [34]. Age (years) was recorded at the time of the physical exam.

Disability status was identified based on four questions from the demographic survey: a condition (1) that limits the ability to walk, run, or play; (2) that has lasted, or is expected to last, 12 months or longer; (3) that requires use of special equipment; and (4) that involves special education or early intervention services [34]. Youth were categorized as “with disability” if the parent answered ‘yes’ to at least one of the four questions. This is consistent with previous analyses of disability using NHANES databases [35,36,37].

Statistical analyses of secondary data were conducted using R (Vienna, Austria) with the “survey” package. NYFS data were downloaded from the National Center for Health Statistics website. All analyses accounted for the 2012 NYFS survey design with sampling weights, primary sampling unit indicators, and stratum variables [17]. The alpha level was set at 0.05 for all analyses. Continuous descriptive statistics were calculated as the weighted mean ± standard error. Categorical descriptive statistics were calculated as a weighted proportion with a 95% confidence interval (CI). Group differences between youth with and without disabilities were examined in three ways. First, raw scores of each musculoskeletal fitness measure were examined with independent t-tests, including Hedges’ g effect size (both weighted and unweighted). Second, categorical fitness scores based on percentiles (i.e., ≤20th percentile vs. >20th percentile [21]) were examined with chi-square tests and Cramer’s V effect size (both weighted and unweighted). Third, differences by BMI category were examined with odds ratios, including 95% CI, based on ordinal logistic regression and binary logistic regression for each BMI category separately. To examine factors associated with low musculoskeletal fitness, binary logistic regression models were used. Separate analyses were conducted for dependent variables of plank, modified pull-ups, and relative grip strength. Each analysis included multiple models to examine the independent (models 1–7) and cumulative (model 8) effects of independent variables, including disability, age, sex, Hispanic origin, meeting physical activity guidelines, engaged in physical activity in last seven days, and BMI category.

## 3. Results

A sample of 1177 youth between the ages of 6 and 15 years (10.5 ± 0.07 years; 49.2% female) were identified from the 2012 NYFS national sample with complete data for the selected variables. Of this sample, 173 youth (15.3% of total sample) were identified with a disability (11.2 ± 0.3 years; 42.6% female). Table 1 provides a descriptive summary of the sample demographics and variables.

Differences in musculoskeletal fitness between youth with and without disabilities were identified for multiple assessments. Table 2 presents the comparison of raw data for each measure. Statistically significant differences between groups were identified for plank (*p* = 0.001) and absolute grip strength (*p* = 0.01), but not modified pull-ups (*p* = 0.13) or relative grip strength (*p* = 0.056). Table 3 presents the comparison of the low-fitness proportion based on the ≤20th percentile of each measure. Statistically significant differences between groups were observed for plank (*p* = 0.02), modified pull-ups (*p* < 0.001), and relative grip strength (*p* < 0.001), but not absolute grip strength (*p* = 0.6). All differences identified were small in magnitude.

YWD also had greater BMI percentiles (72.0 percentile) than youth without disabilities (67.4 percentile; t = 2.2, *p* = 0.04). More than 45% of YWD were categorized as overweight or obese, compared to approximately 36% of youth without disabilities. Table 4 presents odd ratios from the ordinal logistic regression and for YWD across the four weight status categories. According to the ordinal logistic regression, YWD were more likely to be in a higher BMI category (OR = 1.5, *p* = 0.02). When examining the BMI category separately, YWD had 1.4 and 1.3 greater odds than youth without disabilities to be overweight or obese, respectively, but no statistically significant associations were observed (*p* = 0.3).

Logistic regression models are presented in Supplemental Tables S1–S3 for each fitness measure (i.e., plank, modified pull-ups, relative grip strength). Each model examines the associative relationships of low musculoskeletal fitness (≤20th percentile) for each measure. The cumulative models (i.e., model 8) for all three fitness measures are presented in Table 5.

For the plank test, statistically significant independent associations were identified with disability, sex, meeting physical activity guidelines, engaging in physical activity in last seven days, and obese weight status (*p* < 0.05). The cumulative model identified disability (OR = 2.2, *p* = 0.04), sex (OR = 0.4, *p* = 0.001), and obese weight status (OR = 5.9, *p* < 0.001) as significant predictors of plank performance.

For modified pull-ups, statistically significant independent associations were identified with disability, meeting physical activity guidelines, engaging in physical activity in last seven days, and both overweight and obese weight statuses (*p* < 0.05). Cumulative model 8 identified disability (OR = 1.5, *p* = 0.01), sex (OR = 0.7, *p* = 0.03), engagement in physical activity in last seven days (OR = 0.5, *p* < 0.001), overweight status (OR = 2.1, *p* = 0.02), and obese weight status (OR = 7.6, *p* < 0.001) as significant predictors of modified pull-up performance.

Relative grip strength was selected over absolute grip strength due to the significant categorical difference identified between groups (see Table 3). For relative grip strength, statistically significant independent associations were identified with disability, meeting physical activity guidelines, and both overweight and obese weight statuses (*p* < 0.05). Cumulative model 8 identified disability (OR = 2.4, *p* = 0.009), sex (OR = 0.6, *p* = 0.01), meeting physical activity guidelines (OR = 0.6, *p* = 0.01), overweight status (OR = 6.1, *p* < 0.001), and obese weight status (OR = 48.2, *p* < 0.001) as significant predictors of modified pull-up performance.

## 4. Discussion

The purpose of this secondary data analysis was to compare musculoskeletal fitness between youth with and without disabilities in the United States. The use of the 2012 NYFS permitted this examination with sampling techniques purported to be nationally representative.

Low musculoskeletal fitness (≤20th percentile) among YWD was identified in plank (25.2%), modified pull-ups (44.4%), and relative grip strength (30.5%), with statistically significant associations observed between YWD and youth without disabilities across the three measures. After controlling for relevant factors, YWD exhibited greater odds of low musculoskeletal fitness in plank (OR = 2.2), modified pull-ups (OR = 1.5), and relative grip strength (OR = 2.4). The lower musculoskeletal fitness observed in this weighted sample is consistent with previous examinations of fitness between youth with and without disabilities [11,12,13,14,15,16]. For example, Hartman et al. [11] observed lower fitness among youth with borderline/mild intellectual disabilities compared to youth without disabilities. Differences in grip strength (*d* = 0.30–0.46) were very similar to the grip strength disparities observed in the present study.

Additional factors associated with low musculoskeletal fitness were also examined. Weight status had the strongest association with fitness. High rates of overweight (23.2%) and obesity (21.9%) were observed within the sub-sample of YWD. It is well documented that YWD experience health disparities in obesity [38]. In the logistic regression models, obese youth had 44.1-, 7.6-, and 5.9-time greater odds of low grip strength, pull-ups, and plank, respectively, compared to youth with normal weight status. Males were consistently at lower risk of low fitness compared to females, ranging from odds at 0.4 to 0.6 times. Lower musculoskeletal fitness among females has also been consistently reported [18,20,21,23]. Finally, age was not a significant factor in the model for any measure, suggesting that low fitness risk was consistent across the age range of 6–15 years.

There were also a variety of associative relationships identified between low fitness and reported physical activity. For relative grip strength, youth that met physical activity guidelines (e.g., 60 min/seven days per week) had significantly lower odds of low muscular strength compared to youth that did not meet guidelines (OR = 0.6). For modified pull-ups, engagement in physical activity in the last seven days was associated with lower odds of low muscular strength (OR = 0.5). For plank, neither physical activity variable remained statistically significant in the full model. However, both reportedly meeting physical activity guidelines and engaging in physical activity in the last week were independently associated with modified pull-ups and plank. When multiple physical activity measures were associated with low fitness, the stronger predictor was retained in the full model. The association between physical activity levels and more favorable health-related physical fitness is consistent with studies of youth from the general population using both reported [19,20] and objective measures [18] of physical activity.

There are multiple limitations in the secondary analysis that must be acknowledged. First and foremost, specific disabling conditions were not available in the 2012 NYFS. Disability status was based on parental responses to four survey questions and only reflect a proxy of disability. The specific disability diagnosis [39], disability identity [40], and eligibility for special education services [41,42] are important factors that cannot be assumed from the available data. Based on the exclusion criteria used in the 2012 NYFS [27], the sample likely reflects youth with mild impairments. However, the proportion of YWD identified in the sample is consistent with the proportion of youth receiving special education services in 2012 [43]. Second, while the full 2012 NYFS sample can be considered nationally representative, disability status (as operationalized in this secondary analysis) was not part of the sampling design. Third, while the musculoskeletal fitness measures included in NYFS are also included in fitness batteries designed for youth with disabilities (i.e., Brockport Physical Fitness Test [44]), these tests may not be appropriate for all youth with disabilities due to individual aspects of impairment. It is not possible to differentiate the factors associated with disability from low physical fitness in the current analysis. Finally, the 2012 NYFS data are now more than a decade old. This may limit generalizability to youth in the present day. However, until a new national multistage probability survey that directly measures health-enhancing physical fitness of youth is conducted, this dataset remains the best option for examining fitness in a nationally representative sample.

## 5. Conclusions

Significant and meaningful deficits in musculoskeletal fitness were identified among YWD. This was coupled with high rates of overweight and obesity among YWD. These results from a nationally representative sample provide further evidence of the need to provide effective health promotion services and appropriate physical education for this at-risk group.

## Figures and Tables

**Table 1:** Weighted and unweighted descriptive statistics of 2012 NYFS sample: Youth with and without disabilities.

	With Disabilities (n = 173; 15.3%)		Without Disabilities (n = 1004; 84.7%)		Total (n = 1177; 100%)	
Variables	Unweighted Sample Size	Weighted Mean ± SE or Proportion (CI)	Unweighted Sample Size	Weighted Mean ± SE or Proportion (CI)	Unweighted Sample Size	Weighted Mean ± SE or Proportion (CI)
Age, years	173	11.2 ± 0.3	1004	10.3 ± 0.07	1177	10.5 ± 0.07
Sex						
Male, %	97	57.4 (50.9, 64.0)	486	49.6 (47.1, 52.0)	583	50.8 (48.4, 53.0)
Female, %	76	42.6 (36.3, 49.0)	518	50.4 (47.9, 53.0)	594	49.2 (46.8, 52.0)
Hispanic						
Hispanic, %	43	18.2 (8.7, 34.0)	302	23.8 (14.9, 36.0)	345	22.9 (14.2, 35.0)
Non-Hispanic, %	130	81.8 (65.8, 91.0)	702	76.2 (64.4, 85.0)	832	77.1 (65.1, 86.0)
Plank, seconds	173	59.7 ± 2.4	1004	71.7 ± 2.0	1177	69.9 ± 1.8
≤20th Percentile, %	47	25.2 (15.8, 38.0)	138	13.2 (10.5, 16.0)	185	15.0 (12.5, 18.0)
>20th Percentile, %	126	74.8 (62.3, 84.0)	866	86.8 (83.6, 90.0)	992	85.0 (82.0, 88.0)
Modified Pull-Ups, reps	173	4.9 ± 0.5	1004	5.7 ± 0.3	1177	5.6 ± 0.3
≤20th Percentile, %	83	44.4 (37.5, 51.0)	354	32.7 (28.5, 37.0)	437	34.5 (30.4, 39.0)
>20th Percentile, %	90	55.6 (48.6, 62.0)	650	67.3 (62.7, 72.0)	740	65.5 (61.1, 70.0)
Grip Strength, kg	173	46.5 ± 1.4	1004	41.7 ± 0.6	1177	42.4 ± 0.6
≤20th Percentile, %	38	21.2 (14.4, 30.0)	192	19.4 (16.1, 23.0)	230	19.7 (16.2, 23.0)
>20th Percentile, %	135	78.8 (69.9, 86.0)	812	80.6 (76.8, 84.0)	947	80.3 (76.8, 83.0)
Relative Grip Strength, kg/kg ^	173	0.9 ± 0.01	1004	1.0 ± 0.006	1177	1.0 ± 0.005
≤20th Percentile, %	56	30.5 (26.8, 34.0)	183	17.9 (14.9, 21.0)	239	19.9 (17.1, 23.0)
>20th Percentile, %	117	69.5 (65.5, 73.0)	821	82.1 (78.6, 85.0)	938	80.1 (77.0, 83.0)
Body Mass Index Percentile	173	72.0 ± 2.1	1004	67.4 ± 1.2	1177	68.14 ± 1.1
Underweight, %	1	0.8 (0.1, 5.0)	31	3.3 (2.3, 5.0)	32	2.9 (2.1, 4.0)
Normal weight, %	96	54.1 (41.5, 66.0)	605	60.9 (55.9, 66.0)	701	59.9 (55.4, 64.0)
Overweight, %	35	23.2 (12.6, 39.0)	172	17.5 (14.6, 21.0)	207	18.3 (15.2, 22.0)
Obese, %	41	21.9 (16.9, 28.0)	196	18.3 (14.4, 23.0)	237	18.9 (15.3, 23.0)
Physical Activity, days/week	171	6.1 ± 1.3	1002	5.4 ± 0.09	1173	5.5 ± 0.2
Meeting Physical Activity Guidelines						
Yes, %	63	38.4 (30.5, 47.0)	476	46.7 (42.1, 51.0)	539	45.5 (41.8, 49.0)
No, %	108	61.6 (53.0, 70.0)	526	53.3 (48.5, 58.0)	634	54.5 (50.7, 58.0)
Engagement in Physical Activity for Past 7 Days						
Yes, %	127	75.3 (65.6, 83.0)	857	86.0 (81.7, 89.0)	984	84.2 (80.5, 87.0)
No, %	43	24.7 (17.0, 34.0)	147	14.0 (10.7, 18.0)	190	15.6 (12.7, 19.0)

Abbreviations: SE, standard error; CI, 95% confidence interval. ^ Relative grip strength, grip strength kg/body mass kg.

**Table 2:** Comparison of raw musculoskeletal fitness data between youth with and without disabilities.

Musculoskeletal Fitness	Estimated Difference	t	df	*p*	Weighted g	Unweighted g
Plank, seconds	12.0	−4.2	13	0.001 *	0.001	0.3
Modified Pull-Ups, reps	0.8	−1.6	13	0.13	0.0003	0.1
Grip Strength, kg	4.8	3.0	13	0.01 *	−0.0006	−0.3
Relative Grip Strength, kg/kg ^	0.03	−2.1	13	0.056	0.0005	0.1

Abbreviations: t, t-statistic; df, degrees of freedom; *p*, *p*-value; *g*, Hedges’ g effect size. ^ Relative grip strength, grip strength kg/body mass kg. * *p*-Value < 0.05, bolded.

**Table 3:** Comparison of low musculoskeletal fitness (≤20th percentile vs. >20th percentile) between youth with and without disabilities.

Musculoskeletal Fitness	F	ndf	ddf	*p*	Weighted V	Unweighted V
Plank	6.8	1	14	0.02 *	0.0004	0.1
Modified Pull-Ups	29.4	1	14	<0.001 *	0.0009	0.1
Grip Strength	0.2	2	14	0.6	0.0008	0.03
Relative Grip Strength ^	37.0	1	14	<0.001 *	0.001	0.1

Abbreviations: F, F-statistic with Rao and Scott adjustment; ndf, numerator degrees of freedom; ddf, denominator degrees of freedom; *p*, *p*-value; *V*, Cramer’s V effect size. ^ Relative grip strength, grip strength kg/body mass kg. * *p*-Value < 0.05, bolded.

**Table 4:** Odds ratios across body mass index categories between youth with and without disabilities.

	Higher BMI Category			Underweight vs. Other			Normal Weight vs. Other			Overweight vs. Other			Obese vs. Other		
	OR	CI	*p*	OR	CI	*p*	OR	CI	*p*	OR	CI	*p*	OR	CI	*p*
Disability	1.5	1.1, 2.0	0.02 *	0.2	0.03, 1.7	0.2	0.8	0.4, 1.3	0.3	1.4	0.7, 2.9	0.3	1.3	0.9, 1.8	0.3
TD	1			1			1			1			1		

Abbreviations: OR, odds ratio; CI, 95% confidence interval; *p*, *p*-value; TD, typically developing children (without disability). * *p*-Value < 0.05, bolded.

**Table 5:** Logistic regression of plank, modified pull-ups, and grip strength performance (≤20th percentile vs. >20th percentile) between youth with and without disabilities.

Variables		Plank Performance (≤20th Percentile) ^a^	Modified Pull-Ups Performance (≤20th Percentile) ^b^	Relative Grip Strength ^ Performance (≤20th Percentile) ^c^
Disability	With	**2.2** (1.2, 3.8), *p* = 0.04 *	1.5 (1.2, 1.9), *p* = 0.01 *	2.4 (1.5, 3.7), *p* = 0.009 *
	Without	1	1	1
Age		0.9 (0.8, 1.0), *p* = 0.1	1.0 (0.9, 1.0), *p* = 0.2	1.0 (0.9, 1.0), *p* = 0.3
Sex	Male	0.4 (0.3, 0.5), *p* = 0.001 *	0.7 (0.5, 0.9), *p* = 0.03 *	0.6 (0.4, 0.8), *p* = 0.01 *
	Female	1	1	1
Hispanic	Hispanic	0.9 (0.6, 1.3), *p* = 0.5	1.4 (0.9, 2.1), *p* = 0.2	1.0 (0.7, 1.6), *p* = 0.9
	Non-Hispanic	1	1	1
Met PA Guidelines	Yes			0.6 (0.4, 0.8), *p* = 0.01 *
	No			1
Engage in PA Last 7 Days	Yes	0.6 (0.3, 1.0), *p* = 0.08	0.5 (0.4, 0.7), *p* < 0.001 *	
	No	1	1	
Body Mass Index Category	UW	0.9 (0.2, 3.6), *p* = 0.9	0.1 (0.02, 0.8), *p* = 0.07	1.6 (0.4, 5.9), *p* = 0.5
	NW	1	1	1
	OW	1.6 (0.8, 3.0), *p* = 0.2	2.1 (1.4, 3.4), *p* = 0.02 *	6.1 (3.5, 10.5), *p* < 0.001 *
	OB	**5.9** (4.4, 8.0), *p* < 0.001 *	7.6 (4.7, 12.5), *p* < 0.001 *	48.2 (26.1, 88.8), *p* < 0.001 *

Notes: Results are presented as odds ratio (95% confidence interval) *p*-value. Abbreviations: PA, physical activity; UW, underweight; NW, normal weight; OW, overweight; OB, obese. ^ Relative grip strength, grip strength kg/body mass kg. * *p*-Value < 0.05, bolded. ^a^ Logistic regression odds ratio of plank performance and disability status (with/without) adjusted for age, sex (male/female), Hispanic status (yes/no), engagement in physical activity in last 7 days (yes/no), and body mass index category (UW/NW/OW/OB). ^b^ Logistic regression odds ratio of modified pull-up performance and disability status adjusted for age, sex, Hispanic status, engagement in physical activity in last 7 days, and body mass index category. ^c^ Logistic regression odds ratio of relative grip strength performance and disability status adjusted for age, sex, Hispanic status, meeting physical activity guidelines (yes/no), and body mass index category.

## Data Availability

Publicly available datasets were analyzed in this study. These data can be found here: https://wwwn.cdc.gov/nchs/nhanes/search/nnyfs12.aspx (accessed on 21 October 2020).

## Supplementary Materials

The following supporting information can be downloaded at: https://www.mdpi.com/article/10.3390/ijerph22020302/s1: Table S1. Logistic regression of plank performance (≤20th percentile vs. >20th percentile) between youth with and without disabilities; Table S2. Logistic regression of modified pull-ups performance (≤20th percentile vs. >20th percentile) between youth with and without disabilities; Table S3. Logistic regression of relative grip strength performance (≤20th percentile vs. >20th percentile) between youth with and without disabilities.

## Abbreviations

The following abbreviations are used in this manuscript:

YWD	Youth with disabilities
NHANES	National Health and Nutrition Examination Survey
NYFS	National Youth Fitness Survey
