# Supplementary material for: Lower Musculoskeletal Fitness Among Youth with Disabilities, Ages 6 to 15 Years
Source: Int J Environ Res Public Health. 2025 Feb 17;22(2):302. doi: 10.3390/ijerph22020302 (PMC11855659; doi:10.3390/ijerph22020302)
Supplement: Supplementary file 1 [file ijerph-22-00302-s001.zip › ijerph-3426735-supplementary.pdf]

International Journal of
Environmental Research and Public Health

MDPI

Table S1. Logistic regression of plank performance (≤20th percentile vs. >20th percentile) between youth with and without disabilities

Plank Performance (≤20th percentile)																
Variables:		Disability		Age	Sex		Hispanic		Met PA Guidelines		Engage in PA Last 7 Days		Body Mass Index Category			
Model		Yes	No		Male	Female	Hispanic	Non-Hispanic	Yes	No	Yes	No	UW	NW	OW	OB
1	OR	**2.2**	1													
	(CI)	**(1.2, 4.1)**														
	p	**.02***														
2	OR	**2.3**	1	1.0												
	(CI)	**(1.2, 4.2)**		(0.9, 1.0)												
	p	**.02***		.3												
3	OR	**2.4**	1		**0.5**	1										
	(CI)	**(1.3, 4.4)**			**(0.0, 0.7)**											
	p	**.01***			**.002***											
4	OR	**2.2**	1				1.0	1								
	(CI)	**(1.2, 4.1)**					(0.6, 1.6)									
	p	**.03***					1.0									
5	OR	**2.1**	1						**0.7**	1						
	(CI)	**(1.2, 3.9)**							**(0.5, 1.0)**							
	p	**.03***							**.06**							
6	OR	**2.0**	1								**0.5**	1				
	(CI)	**(1.1, 3.5)**									**(0.3, 0.8)**					
	p	**.03***									**.02***					
7	OR	**2.2**	1										0.9	1	1.6	**4.9**

	(CI)	**(1.1, 4.1)**											(0.2, 3.3)		(0.8, 2.9)	**(3.6, 6.7)**
	p	**.04***											.8		.2	**<.001***
8	OR	**2.2**	1	0.9	**0.4**	1	0.9	1			0.6	1	0.9	1	1.6	**5.9**
	(CI)	**(1.2, 3.8)**		(0.8, 1.0)	**(0.3, 0.5)**		(0.6, 1.3)				(0.3, 1.0)		(0.2, 3.6)		(0.8, 3.0)	**(4.4, 8.0)**
	p	**.04***		0.1	**.001***		.5				.08		.9		.2	**<.001***

Abbreviations. OR, odds ratio; CI, 95% confidence interval; *p*, *p*-value; PA, physical activity; UW, underweight; NW, normal weight; OW, overweight; OB, obese.

p*-value **<.05, bolded.

Model 1. Logistic regression odds ratio of the outcome variable, plank performance (≤20th percentile), with the exposure variable, disability status (with/without).

Model 2. Logistic regression odds ratio of plank performance and disability status adjusted for age (continuous).

Model 3. Logistic regression odds ratio of plank performance and disability status adjusted for sex (male/female).

Model 4. Logistic regression odds ratio of plank performance and disability status adjusted for Hispanic status (yes/no).

Model 5. Logistic regression odds ratio of plank performance and disability status adjusted for meeting physical activity guidelines (yes/no).

Model 6. Logistic regression odds ratio of plank performance and disability status adjusted for engagement in physical activity last 7 days (yes/no).

Model 7. Logistic regression odds ratio of plank performance and disability status adjusted for body mass index category (UW, NW, OW, OB).

Model 8. Logistic regression odds ratio of plank performance and disability status adjusted for age, sex, Hispanic status, engagement in physical activity last 7 days, and body mass index category.

Table S2. Logistic regression of modified pull-ups performance (≤20th percentile vs. >20th percentile) between youth with and without disabilities

**Modified Pull-Ups Performance (≤20th percentile)**																
Variables:		Disability		Age	Sex		Hispanic		Met PA Guidelines		Engage in PA Last 7 Days		Body Mass Index Category			
Model		Yes	No		Male	Female	Hispanic	Non-Hispanic	Yes	No	Yes	No	UW	NW	OW	OB
1	OR	**1.6**	1													
	(CI)	**(1.4, 2.0)**														
	p	**<.001***														
2	OR	**1.6**	1	1.0												
	(CI)	**(1.4, 2.0)**		(0.9, 1.1)												
	p	**<.001***		.7												
3	OR	**1.7**	1		0.8	1										
	(CI)	**(1.4, 2.0)**			(0.6, 1.1)											
	p	**<.001***			.2											
4	OR	**1.7**	1				1.5	1								
	(CI)	**(1.4, 2.0)**					(1.0, 2.4)									
	p	**<.001***					.09									
5	OR	**1.6**	1						**0.6**	1						
	(CI)	**(1.3, 1.7)**							**(0.5, 0.8)**							
	p	**<.001***							**<.001***							
6	OR	**1.5**	1								**0.5**	1				
	(CI)	**(1.3, 1.7)**									**(0.3, 0.6)**					
	p	**<.001***									**<.001***					
7	OR	**1.5**	1										0.1	1	**2.1**	**7.1**
	(CI)	**(1.1, 2.1)**											(0.02, 0.8)		**(1.4, 3.2)**	**(4.3, 11.5)**
	p	**<.001***											.05		**.006***	**<.001***
8	OR	**1.5**	1	1.0	**0.7**	1	1.4	1			**0.5**	1	0.1	1	**2.1**	**7.6**
	(CI)	**(1.2, 1.9)**		(0.9, 1.0)	**(0.5, 0.9)**		(0.9, 2.1)				**(0.4, 0.7)**		(0.02, 0.8)		**(1.4, 3.4)**	**(4.7, 12.5)**

	p	**.01***		.2	**.03***		.2				**<.001***		.07		**.02***	**<.001***

Abbreviations. OR, odds ratio; CI, 95% confidence interval; *p*, *p*-value; PA, physical activity; UW, underweight; NW, normal weight; OW, overweight; OB, obese.

p*-value **<.05, bolded.

Model 1. Logistic regression odds ratio of the outcome variable, modified pull up performance (≤20th percentile), with the exposure variable, disability status (with/without).

Model 2. Logistic regression odds ratio of modified pull up performance and disability status adjusted for age (continuous).

Model 3. Logistic regression odds ratio of modified pull up performance and disability status adjusted for sex (male/female).

Model 4. Logistic regression odds ratio of modified pull up performance and disability status adjusted for Hispanic status (yes/no).

Model 5. Logistic regression odds ratio of modified pull up performance and disability status adjusted for meeting physical activity guidelines (yes/no).

Model 6. Logistic regression odds ratio of modified pull up performance and disability status adjusted for engagement in physical activity last 7 days (yes/no).

Model 7. Logistic regression odds ratio of modified pull up performance and disability status adjusted for body mass index category (UW, NW, OW, OB).

Model 8. Logistic regression odds ratio of modified pull up performance and disability status adjusted for age, sex, Hispanic status, engagement in physical activity last 7 days, and body mass index category.

Table S3. Logistic regression of relative grip strength performance (≤20th percentile vs. >20th percentile) between youth with and without disabilities

**Relative Grip Strength^ Performance (≤20th percentile)**																
Variables:		Disability		Age	Sex		Hispanic		Met PA Guidelines		Engage in PA Last 7 Days		Body Mass Index Category			
Model		Yes	No		Male	Female	Hispanic	Non-Hispanic	Yes	No	Yes	No	UW	NW	OW	OB
1	OR	**2.0**	1													
	(CI)	**(1.6, 2.5)**														
	p	**<.001***														
2	OR	**2.0**	1	1.0												
	(CI)	**(1.5, 2.5)**		(0.9, 1.1)												
	p	**<.001***		0.7												
3	OR	**2.0**	1		0.9	1										
	(CI)	**(1.6, 2.6)**			(0.7, 1.2)											
	p	**<.001***			0.4											
4	OR	**2.0**	1				1.2	1								
	(CI)	**(1.6, 2.6)**					(0.9, 1.7)									
	p	**<.001***					0.2									
5	OR	**1.9**	1						**0.5**	1						
	(CI)	**(1.5, 2.5)**							**(0.4, 0.7)**							
	p	**<.001***							**<.001***							
6	OR	**1.9**	1								0.7	1				
	(CI)	**(1.4, 2.6)**									(0.4, 1.1)					
	p	**.001***									0.1					
7	OR	**2.2**	1										1.6	1	**6.0**	**44.1**
	(CI)	**(1.6, 3.2)**											(0.4, 6.2)		**(3.4, 10.6)**	**(24.4, 79.5)**
	p	**.001***											0.5		**<.001***	**<.001***
8	OR	**2.4**	1	1.0	**0.6**		1.0	1	**0.6**	1			1.6	1	**6.1**	**48.2**
	(CI)	**(1.5, 3.7)**		(0.9, 1.0)	**(0.4, 0.8)**		(0.7, 1.6)		**(0.4, 0.8)**				(0.4, 5.9)		**(3.5, 10.5)**	**(26.1, 88.8)**

	p	**.009***		0.3	**0.01***		0.9		**0.01***				0.5		**<.001***	**<.001***

Abbreviations. OR, odds ratio; CI, 95% confidence interval; *p*, *p*-value; PA, physical activity; UW, underweight; NW, normal weight; OW, overweight; OB, obese.

^ Relative grip strength, grip strength kg / body mass kg.

p*-value **<.05, bolded.

Model 1. Logistic regression odds ratio of the outcome variable, relative grip strength performance (≤20th percentile), with the exposure variable, disability status (with/without).

Model 2. Logistic regression odds ratio of relative grip strength performance and disability status adjusted for age (continuous).

Model 3. Logistic regression odds ratio of relative grip strength performance and disability status adjusted for sex (male/female).

Model 4. Logistic regression odds ratio of relative grip strength performance and disability status adjusted for Hispanic status (yes/no).

Model 5. Logistic regression odds ratio of relative grip strength performance and disability status adjusted for meeting physical activity guidelines (yes/no).

Model 6. Logistic regression odds ratio of relative grip strength performance and disability status adjusted for engagement in physical activity last 7 days (yes/no).

Model 7. Logistic regression odds ratio of relative grip strength performance and disability status adjusted for body mass index category (UW, NW, OW, OB).

Model 8. Logistic regression odds ratio of relative grip strength performance and disability status adjusted for age, sex, Hispanic status, meeting physical activity guidelines, and body mass index category
